# Screening the immune-related circRNAs and genes in mice of spinal cord injury by RNA sequencing

**DOI:** 10.3389/fimmu.2022.1060290

**Published:** 2022-11-21

**Authors:** Yongjin Li, Baobao Wang, Wenzhi Sun, Chao Kong, Guowang Li, Xiaolong Chen, Shibao Lu

**Affiliations:** ^1^ Department of Orthopedics, Xuanwu Hospital, Capital Medical University, Beijing, China; ^2^ Graduate School, Tianjin Medical University, Tianjin, China

**Keywords:** spinal cord injury, RNA-sequencing, circular RNA, immune, inflammation

## Abstract

Spinal cord injury (SCI) is a pathological condition that leading to serious nerve damage, disability and even death. Increasing evidence have revealed that circular RNAs (circRNAs) and mRNA are widely involved in the regulation of the pathological process of neurological diseases by sponging microRNAs (miRNAs). Nevertheless, the potential biological functions and regulatory mechanisms of circRNAs in the subacute stage of SCI remain unclear. We analyzed the expression and regulatory patterns of circRNAs and mRNAs in SCI mice models using RNA-sequencing and bioinformatics analysis. A total of 24 circRNAs and 372 mRNAs were identified to be differentially expressed. Then we identifying the immune-related genes (IRGs) from them. The protein-protein interaction network were constructed based on the STRING database and Cytoscape software. Furthermore, Go and KEGG enrichment analysis were conducted to predict the functions of the IRGs and host genes of DECs. These findings will contribute to elucidate the pathophysiology of SCI and provide effective therapeutic targets for SCI patients.

## Introduction

Spinal cord injury (SCI) is a life-shattering central nervous system injury with high disability rate and mortality rate, which is known to bring serious social-economic burden around the world (1, 2). It has been reported that the annual incidence of SCI in different countries ranges from 11.5 to 57.8 cases per million people (3, 4), and these patients face a heavy economic burden and a significantly reduced quality of life because SCI is often accompanied by permanent neurological impairment and various complications (1, 5). At present, the etiological classification of SCI is roughly divided into two categories: traumatic and nontraumatic injuries (2). The traffic and sports accident, violence and falls are the predominant causes of traumatic SCI (2, 3), while nontraumatic injuries is mainly caused by infection and tumor (2, 6). Some scholars have divided the pathophysiology of SCI into primary mechanism and secondary mechanism. SCI often start with primary mechanical injury, the structural integrity of the spinal cord is damaged under severe impact or continuous compression, which is usually the most important factor to determine the severity of SCI; then gradually progress into secondary SCI with a series of endogenous molecular pathological processes, such as the abnormally elevated inflammatory response, oxidative stress, and immune response (7–9). Secondary SCI can be divided into acute, subacute, and chronic stages, with the subacute stage lasting 2 days to 2 weeks after SCI (10, 11). The subacute stage of SCI is a potentially critical time point for further study of SCI repair, which may play a significant role in the secondary SCI (10, 11). Current treatments are limited and cannot completely recover the function of the spinal cord (12, 13), and the effective therapeutic strategies to avoid secondary SCI are still lacking. Therefore, it is very necessary to comprehensively and deeply understand the molecular pathological mechanisms following secondary SCI (especially in the subacute stage) to explore new therapeutic methods that promote the functional recovery of spinal cord.

Non-coding RNAs (ncRNAs) are a group of RNAs that regulate messenger RNAs (mRNAs), and its family includes circular RNAs (circRNAs), long-chain ncRNAs (lncRNAs), and microRNAs (miRNAs), most of which cannot encode proteins and can regulate cellular physiology and functions (14). CircRNA is a major regulatory single-stranded ncRNA and an important post-transcriptional regulatory element without 5’ to 3’ polarity and polyadenylated tail, its 5’ and 3’ ends are linked together to form a covalently closed circular structure, this special structure endows it with high stability and is not easily degraded by exonuclease, which can serve as novel diagnostic and prognostic biomarkers in many diseases (15–17). Accumulating evidence have suggested that miRNAs can accelerate target gene mRNAs degradation prevent its translation and reducing its expression by directly binding to the 3’ untranslated region (3’UTR) of mRNAs in the cytoplasm (18, 19). As competing endogenous RNA (ceRNA), circRNAs were reported to be widely involved in regulating the pathological process after SCI by targeting miRNAs-mRNA pathway, such as inflammatory response, oxidative stress, cell death, autophagy, neuronal migration, and adhesion (20–28). Wang et al. (21) verified that the circPlek/miR-135b-5p/TGF-βR1 signaling axis is involved in the activation of fibroblasts and fibrous scar formation in the subacute stage of SCI. Nevertheless, the potential biological functions and regulatory mechanisms of circRNAs in the subacute stage of SCI remain unclear.

In this study, we construct a contusion SCI mouse model. High-throughput RNA-sequencing (RNA-seq) technology was utilized to measure the circRNAs and mRNAs expression profile on 4th day after SCI in mice. We identified 24 differentially expressed circRNAs (DECs) and 372 differentially expressed genes (DEGs). Then we identifying the immune-related genes (IRGs) from them. In addition, we conducted Go and KEGG enrichment analysis to predict the functions of the IRGs and host genes of DECs.

## Material and methods

### Construction of mouse SCI model

We purchased six C57BL/6 male mice (8 weeks, weights 20-25g) from the Institute of Radiation Medicine, Chinese Academy of Medical Sciences. They were randomly divided into 2 groups, namely control (n = 3) and SCI group (n = 3). The mice were anesthetized through intraperitoneal injection of 90 mg/kg ketamine and 10 mg/kg xylazine. Three control mice underwent a laminectomy at the T10 level without injury to the spinal cord, the other three experimental mice were placed on the NYU IMPACTOR MODEL-II spinal cord hitter platform after laminectomy at the T10 level, and the T10 spinal cord was hit with a hitting head weighing 10 grams to construct a SCI model. All the six mice were killed on the 4th day after surgery, and the spinal cord tissue about 4 cm long around the T10 level were collected for high-throughput RNA-sequencing. All mice procedures were in consistent with international and national animal care and ethical guidelines and have been approved by the institutional animal welfare committee.

### Construction of sequencing library

The TRlzol kit (B511311, Sangon, China) was used to extract total RNAs from spinal cord tissues. Then we added RNase-free DNase I to remove genomic DNA. The ribosomal RNA was removed from total RNA with Ribo-off rRNA depletion kit (Nanjing, China). To measure RNA quality and purity, we used NanoPhotometer ^®^ spectrophotometer (IMPLEN, CA, USA) and an Agilent 2100 bioanalyzer (Agilent Technologies, CA, USA) for detection. Subsequently, VAHTSTM mRNA-seq V2 Library Prep Kit for Illumina^®^ was used to construct sequencing libraries according to manufacturer’s suggestions. High-quality RNA was further purified by a RNeasy mini kit (QIAGEN, GmBH, Germany). Finally, a HiSeq Xen sequencer (Illumina, San Diego, CA) was used for pair-end sequencing of the libraries.

### Analysis of circRNAs and mRNAs expression profile

RNA sequencing program were conducted by Sangon Biotechnology Company (Shanghai, China). The quality of the sequencing raw data was assessed by FastQC, and we then performed mass clipping using Trimmomatic to get relatively accurate valid data. The data were aligned to the reference genome using bwa, and circRNAs were then identified by CIRI2. The origination of circRNAs were determined based on their location information and known gene annotations using BEDtools. The expression of circRNAs was calculated by the reads per kilobase of transcript per million mapped reads (RPKM) formula based on BSJ reads. The expression of mRNAs was calculated by the transcripts per kilobase of exon model per million mapped reads (TKM) formula. The data processing were conducted using the R software package. The differentially expressed circRNAs (DECs) and differentially expressed mRNAs (DEMs) were analyzed using DESeq2 software. The screening criteria for DECs was |log_2_ fold-change (FC)| > 2 and P value < 0.05; and the screening criteria for DEMs was |log_2_ FC| > 5 and P.adj value < 0.01. Hierarchical Clustering and volcano plot were conducted to display the distinguishable circRNAs and mRNAs expression pattern among samples.

### Identifying the IRGs related to SCI

We obtained the IRGs from the online immunology database and analysis portal (immport, https://www.immport.org/home) (29). Venn analysis was performed to select IRGs related to SCI through merging the DEGs and IRGs.

### The pathway enrichment analysis

Gene Ontology (GO) functional annotations and Kyoto Encyclopedia of Genes and Genomes (KEGG) pathway enrichment analysis were conducted to predict the potential roles of the host genes of DECs. Briefly, GO analysis was applied to elucidate genetic regulatory networks of interest by forming hierarchical categories according to the BP, CC, and MF aspects of the differentially expressed genes (http://www.geneontology.org). Pathway analysis was performed to explore the significant pathways of the host genes of DECs according to KEGG (http://www.genome.jp/kegg/). The analytical results were visualized by clusterProfiler package in R (30). A value of p <0:05 was set as a statistically significant difference.

### Construction protein-protein interaction network and identification of hub genes

The Search Tool for the Retrieval of Interacting Genes (STRING) database (https://cn.string-db.org/) was used to evaluate PPIs in functional protein association networks (31). Then we imported the DEGs into the multiple protein section of STRING database and set the organisms as “Mus musculus” to generate the PPI network data. The “required score” was set at > 0.4. Subsequently, the PPI data was imported into Cytoscape (https://cytoscape.org/) software to obtain the top 10 genes as hub genes using the Maximal Clique Centrality (MCC) method of the cytoHubba plugin (32) in Cytoscape.

## Results

### Determining the reasonable for the samples selection and grouping

**Figure 1:**
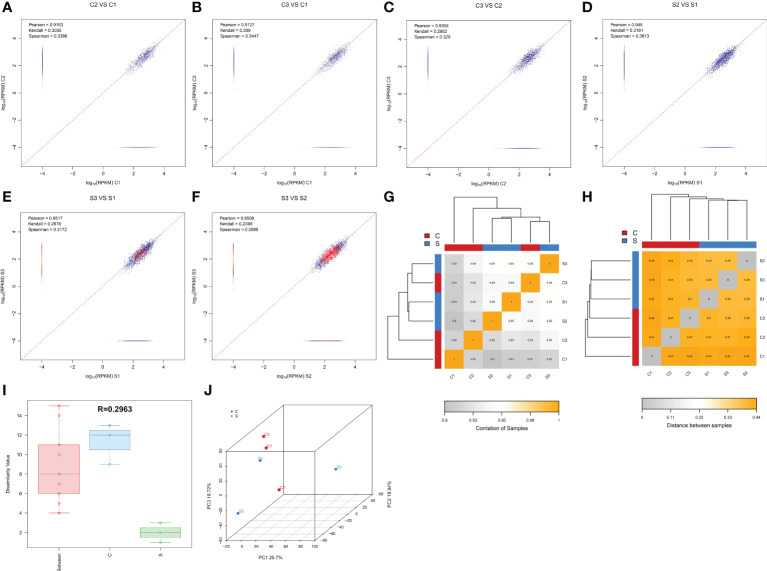
Determining the reasonable for the samples selection. **(A–F)** Duplicate correlation check scatter plot. The horizontal and vertical coordinates are the log_10_ (RPKM) values for the two samples, respectively. Three correlation indices are calculated in the figure: pearson, kendall, and spearman. The more similar the samples are, the closer the similarity index is to 1, and most of the points will be concentrated near the diagonal. **(G)** Inter-sample correlation analysis Heatmap. The color block represents the correlation index value. The grayer the color, the lower the correlation index among samples. The yellower the color, the higher the correlation index. **(H)** Heatmap of distance between samples. The color block represents the distance value. The grayer the color, the closer the distance between samples and the higher the similarity. The yellower the color, the farther the distance is. **(I)**: Anosim between-group similarity analysis boxplot. The boxes on the left refer to the between-group differences, and the middle and right boxes represent the within-group differences of the normal and SCI groups, respectively. The R value ranges from -1 to +1, with an R value closer to 1 indicating greater between-group differences than within-group differences. **(J)** Principal component analysis diagram, the horizontal and vertical coordinates reflect the relative distance between samples. Red points indicate control group, whereas green points indicate SCI group.

This study collected 6 spinal cord tissues from normal and SCI mice for circRNA-sequencing. In order to ensure more reliable results of the bioinformatics analysis of the sequencing results, we carried out the correlation analysis of expression levels between samples, which is an important indicator to detect the reliability of the experiment and whether the sample selection is reasonable. As shown in **Figures 1A–F**, the pearson correlation coefficient among samples is close to 1, indicating that the expression patterns among samples are similar. The inter-sample correlation analysis Heatmap (**Figure 1G**) and the heatmap of distance between samples (**Figure 1H**) further revealed the high similarity among samples. Moreover, There was a greater differences between intra-group and inter-group, suggesting this grouping is meaningful (**Figure 1I**). Principal Component Analysis (PCA) can be used to reflect the difference and distance between samples. The closer the distance in the PCA diagram, the more similar the sample composition. **Figure 1J** presented that there was a relatively consistent similarity between samples and the grouping is reasonable. In addition, the uniformity of each group of repeated samples is good, and the following difference analysis can be carried out (**Figure 1J**). Taken together, these results unveiled that the samples selection and grouping meet experimental requirements.

### The characteristics and expression profiles of circRNA in the subacute stage after SCI in mice

**Figure 2:**
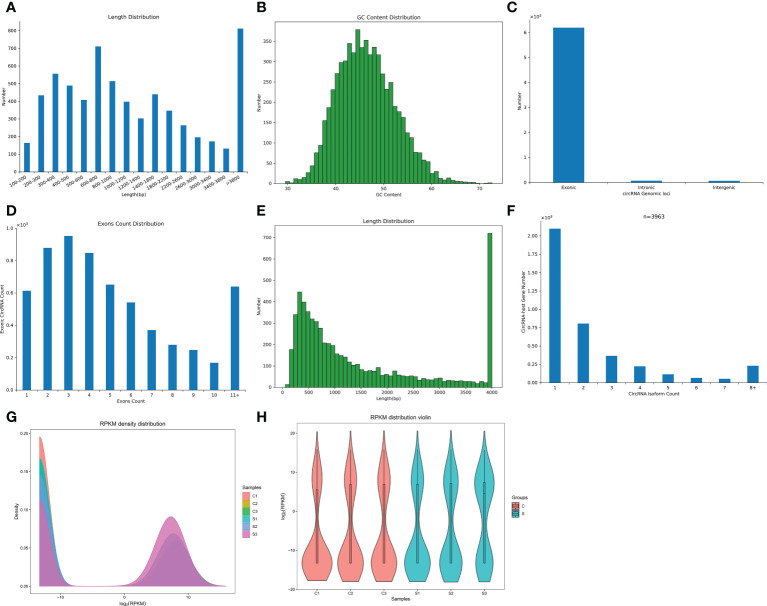
The characteristics and expression profiles of circRNA in the subacute stage after SCI in mice. **(A)** The length distribution of circRNAs. **(B)** The GC content distribution of circRNAs. **(C)** The statistical diagram of circRNAs sources. **(D)** The exons count distribution of circRNAs. **(E)**: The exons length distribution of circRNAs. **(F)** The distribution of circRNA host genes numbers and circRNA isoform count. **(G)** CircRNA expression density curve. The horizontal axis represent the log (RPKM) value, the higher the value, the higher the circRNA expression level; the vertical axis represent the corresponding relative density value, that is, the number of circRNAs on the horizontal axis/the total number of circRNAs. Each color represents a sample, the area of each region is 1, and the peak of the density curve represents the region with the most concentrated circRNA expression in the entire sample. **(H)** Violin plot of circRNA expression calculated using RPKM Reads. The horizontal axis represent the sample name, and the vertical axis represent the log (RPKM) value. The width of each violin reflects the number of circRNAs at that expression levels.

We found a total of 6341 circRNAs by circRNA sequencing. The length distribution and GC content distribution of them was shown in **Figures 2A, B**. Among them, circRNAs with 600-800 and more than 3800 base pairs and 45 GC content accounted for the largest proportion. Then, we evaluated the proportion of circRNAs derived from exons, introns, and intergenic regions in the identified circRNAs, **Figure 2C** displayed that the vast majority of circRNAs were derived from gene exons regions, and a few circRNAs were derived from gene introns and intergenic regions. Most exonic circRNAs count were between 2-4 (**Figure 2D**). Interestingly, most exonic circRNAs were 4000 base pairs in length (**Figure 2E**). Zheng et al (33). reported that there was often a predominantly expressed circRNA isoform from one gene locus, our study also observed that most of the host genes produced one isoform (**Figure 2F**).

FPKM denoted the number of reads per kilobase length from a circRNA per million reads, which can be used to count the abundance of circRNA expression in different samples. RPKM method can eliminate the influence of circRNA length and sequencing quantity difference on the calculation of circRNA expression, and thus FPKM value represented the circRNA expression level. The circRNAs expression abundance in different samples were shown with density curve plot (**Figure 2G**) and violin plot (**Figure 2H**) using RPKM method, suggesting that the circRNAs were differential expression in the subacute phase after SCI in mice.

### The circRNAs were differentially expressed in the subacute stage after SCI in mice

**Figure 3:**
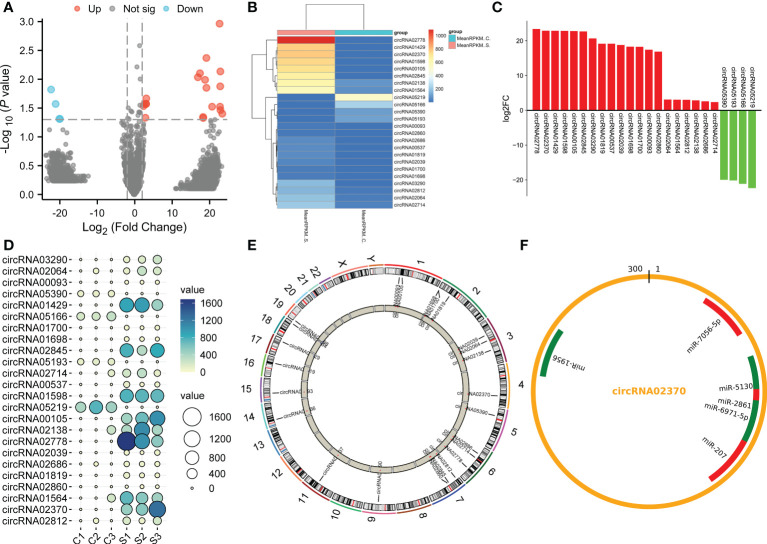
The circRNAs were differentially expressed in the subacute stage after SCI in mice. **(A)** Volcano plot. Green points indicate down-regulation (left side), gray points indicate undifferential expression (middle), and red points indicate up-regulation (right side). **(B)** Hierarchical clustering. with rows indicating DECs and columns indicating samples. **(C)** Bar plot shown the top 20 upregulated and 4 downregulated DECs in SCI tissues. The X-axis represents miRNA and the Y-axis represents log2 fold change. Red means up-regulation, above the X-axis; green means down-regulation, below the X-axis. **(D)** Ballon plot. The size of the circle shows the size of each value in the matrix, and the size of the value is represented by the shade of color. **(E)** The distribution of DECs on chromosomes. **(F)** CircRNA targeted miRNA distribution circle. The orange circle represents circRNA, and the red segment represents miRNA.

To compare differences in circRNA expression between the control group and the SCI group, we conducted circRNA differential expression analysis. The significance threshold was set as |log2 (fold-change)| > 2 and P value < 0.05. Through the analysis of the sequencing results, we identified 24 DECs, of which 20 DECs were up-regulated and 4 DECs were down-regulated in the subacute stage after SCI in mice (**Figure 3A**). Hierarchical clustering analysis further unveiled the 24 DECs between normal and SCI tissues (**Figure 3B**). According to the log2 (fold-change), the top 20 up-regulated and 4 down-regulated DECs in SCI tissues were visualized in **Table 1** and **Figure 3C**, of which circRNA02778 and circRNA02370 were top up-regulated and circRNA05219 was top down-regulated DECs in SCI tissues. Furthermore, the specific expression of 24 DECs in 3 normal and 3 SCI tissues were further shown with ballon plot (**Figure 3D**). To explore the origin of these DECs, the distribution of DECs on chromosomes were analyzed. The results unveiled that these DECs were distributed on the human chromosomes 1-7, 9,11, and 14-19 (**Figure 3E**). To explore the interaction between circRNA and miRNA, miRanda database and the miRBase sequence of the mice are used to predict the target miRNA of circRNA. Venn diagram shown the mmu-miR-7056-5p, mmu-miR-5130, mmu-miR-2861, mmu-miR-6971-5p, mmu-miR-207, and mmu-miR-1956 were the downstream targets of circRNA02370 (**Figure 3F**).

**Table 1:** The expression profile of DEC sin SCI tissue sin mice.

circRNA	log2FC	P Value	Expression
circRNA02778	23.378252	0.039683944	up
circRNA02370	22.88608881	0.034350749	up
circRNA01429	22.86905921	0.007337619	up
circRNA01598	22.8284641	0.01330039	up
circRNA00105	22.77940422	0.029959172	up
circRNA02845	22.65361058	0.001087619	up
circRNA03290	20.65823853	0.029959172	up
circRNA01819	19.14461664	0.014108052	up
circRNA00537	19.13661319	0.004282085	up
circRNA02039	18.77929156	0.010261397	up
circRNA01698	18.29319659	0.047053684	up
circRNA01700	18.24867342	0.045271645	up
circRNA00093	17.42548821	0.007869117	up
circRNA02860	16.8670717	0.009248431	up
circRNA02064	3.082454738	0.021587718	up
circRNA01564	3.032968379	0.02677553	up
circRNA02812	3.013834348	0.026814454	up
circRNA02138	2.851016978	0.046616704	up
circRNA02686	2.589680564	0.026961922	up
circRNA02714	2.350170435	0.029230947	up
circRNA05390	-19.9568273	0.048811362	down
circRNA05193	-20.13556347	0.048811362	down
circRNA05166	-21.12786911	0.026879289	down
circRNA05219	-22.34705232	0.015115009	down

### The genes were differentially expressed in the subacute stage after SCI in mice

**Figure 4:**
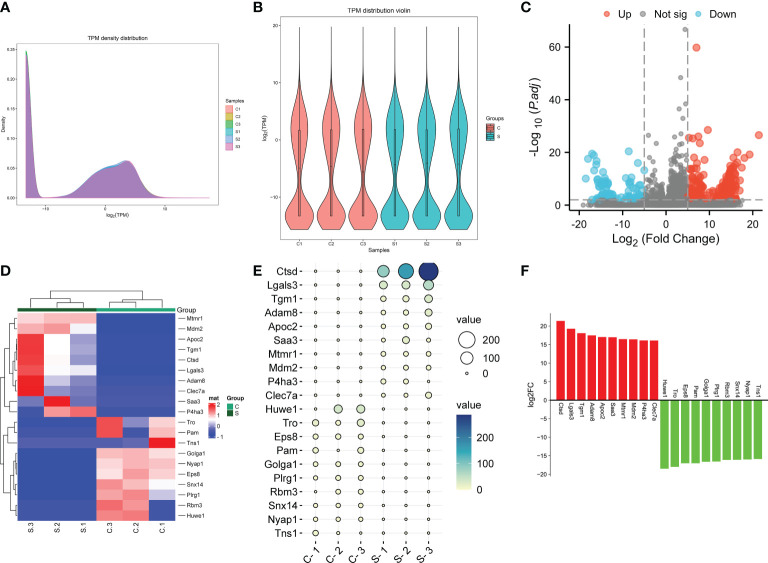
The genes were differentially expressed in the subacute stage after SCI in mice. **(A)** Gene expression density curve. The horizontal axis represent the log (TPM) value, the higher the value, the higher the gene expression level; the vertical axis represent the number of genes on the horizontal axis/the total number of genes. **(B)** Violin plot of gene expression calculated using TPM. The horizontal axis represent the sample name, and the vertical axis represent the log (TPM) value. The width of each violin reflects the number of genes at that expression levels. **(C)** Volcano plot. Green points indicate down-regulation (left side), gray points indicate undifferential expression (middle), and red points indicate up-regulation (right side). **(D)** Hierarchical clustering shown the top 10 upregulated and top 10 downregulated genes. with rows indicating DECs and columns indicating samples. **(E)** Ballon plot. The size of the circle shows the size of each value in the matrix, and the size of the value is represented by the shade of color. **(F)** Bar plot shown the top 10 upregulated and top 10 downregulated genes in SCI tissues. The X-axis represents gene and the Y-axis represents log2 fold change. Red means up-regulation, above the X-axis; green means down-regulation, below the X-axis.

The expression of genes in the subacute stage after SCI was calculated by the TKM method. Based on this method, the genes expression abundance in different samples were displayed using density curve plot (**Figure 4A**) and violin plot (**Figure 4B**). Differential genes expression analysis uncovered that 372 DEGs after SCI, among which 274 DEGs were up-regulated and 98 DEGs were down-regulated based on the standard of |log2 fold-change| > 5 and P.adj value < 0.01 (**Figure 4C**). Moreover, the specific expression of top 10 up-regulated and down-regulated DEGs were presented with hierarchical clustering, ballon plot, and Bar plot (**Figures 4D–F**). The most significantly up- and down-regulated genes were Ctsd and Huwe1, respectively (**Figures 4C–F**).

### Identifying the IRGs related to SCI

**Figure 5:**
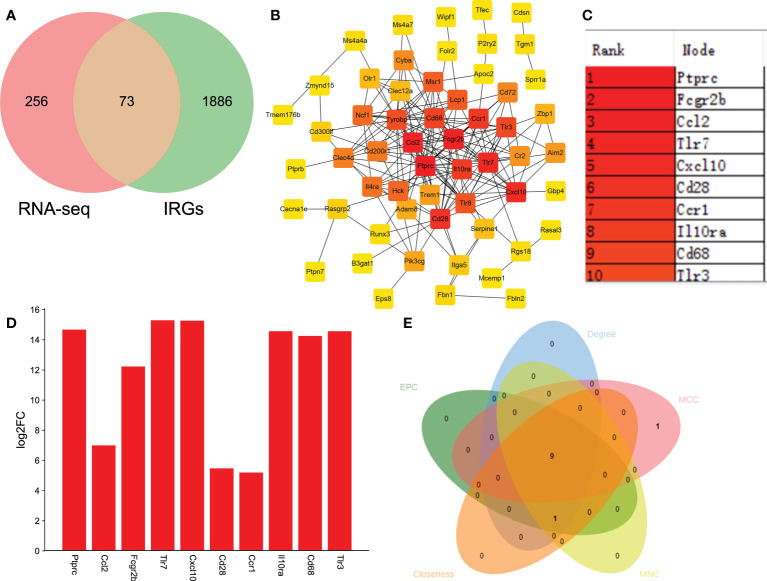
Identifying the IRGs related to SCI. **(A)** Venn analysis to select the overlapping genes between RNA-seq and immune-related genes (IRGs). **(B)** Identifying the key hub genes *via* constructing the protein-protein interaction network and further visualizing using Cytoscape software. **(C)** The top 10 hub genes from PPI network. **(D)** Bar plot shown the top 10 IRGs related to SCI. The X-axis represents gene and the Y-axis represents log2 fold change. Red means up-regulation, above the X-axis. **(E)** MCC, MNC, Degree, EPC, and Closeness algorithms were used to verify the obtaining 10 hub genes in Cytoscape software.

Immune and neuroinflammation exert an important role after SCI (34). Thus, we selected the IRGs related to SCI *via* the intersection of DEGs and IRGs. A total of 73 DEGs were identified as IRGs (**Figure 5A**). To further identify the key hub genes, we constructed a PPI network of 73 DEGs using the STRING database. Then we used the MCC algorithms of the cytoHubba plugin in Cytoscape to obtain the top 10 genes as hub genes (**Figures 5B, C**). The top 10 genes were listed as follows: Ptprc, Fcgr2b, Ccl2, Tlr7, Cxcl10, Cd28, Ccr1, Il10ra, Cd68, and Tlr3 (**Figure 5C**). Interestingly, they all significantly upregulated after SCI (**Figure 5D**). Additionally, we used MCC, MNC, Degree, EPC, and Closeness algorithms to verify the obtaining 10 hub genes in Cytoscape software. A total of 9 overlapping genes (except Ccr1) were predicted *via* Venn diagram (**Figure 5E**), suggesting that Ptprc, Fcgr2b, Ccl2, Tlr7, Cxcl10, Cd28, Il10ra, Cd68, and Tlr3 were the key IRGs.

### Go functional and KEGG pathway enrichment analysis of the host genes of DECs

**Figure 6:**
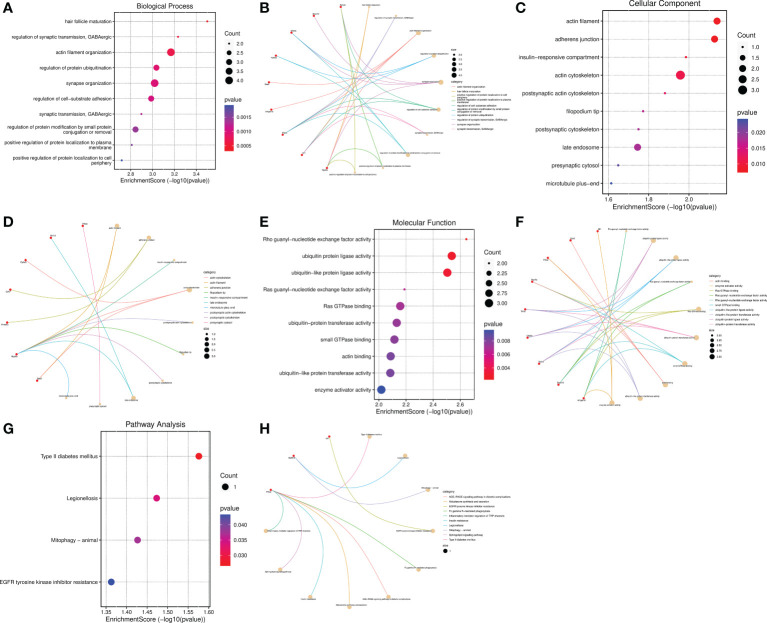
Functional and pathway enrichment analysis of the host genes of DECs. **(A–F)** Bubble diagram and cnetplot showing the host genes of DECs enriched in the different GO categories, including BP, CC, and MF. **(G, H)** KEGG pathway enrichment analysis of the host genes of DECs.

In view of circRNAs are derived from precursor (pre)-mRNAs and formed by back-splicing, namely a downstream 5′ splice site is covalently binded with an upstream 3′ splice site (15–17, 35). Moreover, the abundance of circRNAs are negatively correlated with their linear host genes mRNAs because of there is a competition between circRNA biogenesis and pre-mRNA splicing (36). Thus, whether the host genes of DECs are involved in the regulation of SCI is a meaningful issue worth studying. Subsequently, Go functional and KEGG pathway enrichment analyses were conducted to investigate the potential biological functions of their host genes. GO function annotations included terms in the biological process (BP), molecular function (MF), and cellular component (CC) categories. The top 10 BP terms encompassed hair follicle maturation, regulation of synaptic transmission GABAergic, actin filament organization, regulation of protein ubiquitination, synapse organization, regulation of cell-substrate adhesion, synaptic transmission, GABAergic, regulation of protein modification by small protein conjugation or removal, positive regulation of protein localization to plasma membrane, and positive regulation of protein localization to cell periphery(**Figures 6A, B**). The enriched top 10 CC were actin filament, adherens junction, insulin-responsive compartment, actin cytoskeleton, postsynaptic actin cytoskeleton, filopodium tip, postsynaptic cytoskeleton, late endosome, presynaptic cytosol, and microtubule plus-end (**Figures 6C, D**). The MF analysis unveiled that the host genes of DECs were significantly enriched in the following terms: Rho guanyl-nucleotide exchange factor activity, ubiquitin protein ligase activity, ubiquitin-like protein ligase activity, Ras guanyl-nucleotide exchange factor activity, Ras GTPase binding, ubiquitin-protein transferase activity, small GTPase binding, actin binding, ubiquitin-like protein transferase activity, and enzyme activator activity (**Figures 6E, F**). The cneplots shown the enrichment of the host genes of DECs in each pathway (**Figures 6B, D, F**).

The enriched top 10 KEGG pathways included the Prkce-mediated Type II diabetes mellitus、Fc gamma R-mediated phagocytosis AGE-RAGE signaling pathway in diabetic complications Aldosterone synthesis and secretion、Insulin resistance、Sphingolipid signaling pathway and Inflammatory mediator regulation of TRP channels, Bcl2l13-mediated Legionellosis and Mitophagy, and Nf1-mediated EGFR tyrosinekinase inhibitor resistance (**Figures 6G, H**). Among them, the pathways of Type II diabetes mellitus, Legionellosis, Mitophagy, and EGFR tyrosinekinase inhibitor resistance were statistically significant (P value<0.05) (**Figure 6G**). Taken together, these results suggested that the host genes of DECs possibly regulate synapse function, inflammatory response, and mitophagy, etc.

### Go functional and KEGG pathway enrichment analysis of the IRG-related DEGs

**Figure 7:**
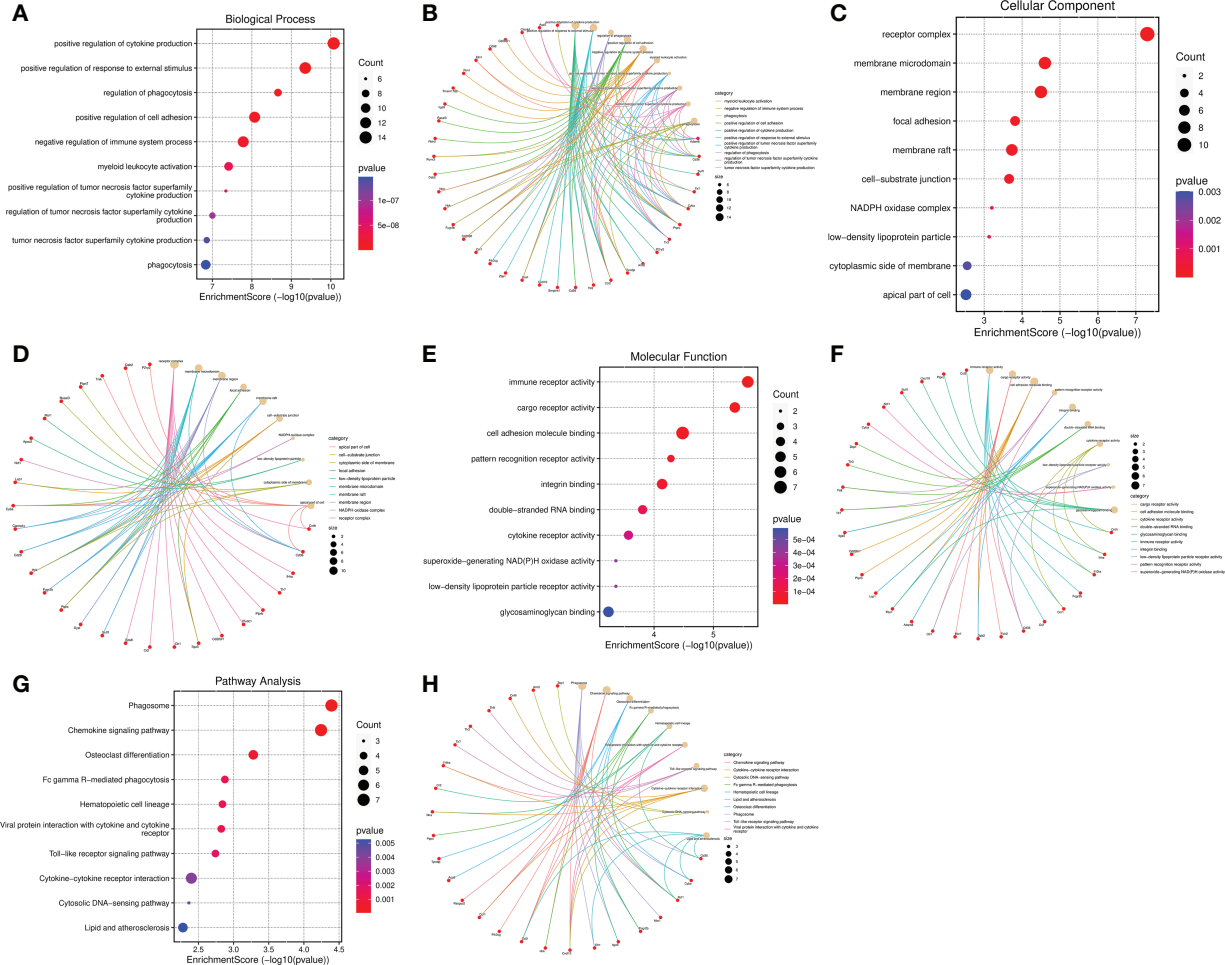
Functional and pathway enrichment analysis of the IRGs related to SCI. **(A–F)** Bubble diagram and cnetplot showing the host genes of DECs enriched in the different GO categories, including BP, CC, and MF. **(G, H)** KEGG pathway enrichment analysis of the host genes of DECs.

The top 10 BP terms including positive regulation of cytokine production, positive regulation of response to external stimulus, regulation of phagocytosis, positive regulation of cell adhesion, negative regulation of immune system process, myeloid leukocyte activation, positive regulation of tumor necrosis factor superfamily cytokine production, regulation of tumor necrosis factor superfamily cytokine production, tumor necrosis factor superfamily cytokine production, and phagocytosis (**Figures 7A, B**). The enriched CC were receptor complex, membrane microdomain, membrane region, focal adhesion, membrane raft, cell-substrate junction, NADPH oxidase complex, low-density lipoprotein particle, cytoplasmic side of membrane, and apical part of cell (**Figures 7C, D**). The MF were significantly enriched in the following terms: immune receptor activity, cargo receptor activity, cell adhesion molecule binding, pattern recognition receptor activity, integrin binding, double-stranded RNA binding, cytokine receptor activity, low-density lipoprotein particle receptor activity, superoxide-generating NAD(P)H oxidase activity, and glycosaminoglycan binding (**Figures 7E, F**). The cneplots shown the enrichment of the host genes of DECs in each pathway (**Figures 7B, D, F**). The KEGG enrichment analysis results were described as follows: Phagosome, Chemokine signaling pathway, Osteoclast differentiation, Fc gamma R-mediated phagocytosis, Hematopoietic cell lineage, Viral protein interaction with cytokine and cytokine receptor, Toll-like receptor signaling pathway, Cytokine-cytokine receptor interaction, Cytosolic DNA-sensing pathway, and Lipid and atherosclerosis (**Figures 7G, H**).

## Discussion

In the past decade, genetic regulation of the occurrence and progression of diseases has received increasing attention. The occurrence of a large number of DECs and DEGs after SCI is the pathological features of SCI (20–28), but the mechanism by which circRNAs-mediated the pathological process in the subacute stage after SCI has not been reported yet. In the present study, the circRNAs and mRNAs expression profiles were sequenced by high-throughput sequencing technology in the mice model of the subacute stage after SCI, and the characteristics and expression levels of DECs and DEGs were analyzed. Through the bioinformatics analysis, we found 20 DECs and 274 DEGs were up-regulated as well as 4 DECs and 98 DEGs were down-regulated after SCI in mice. Among them, circRNA02778 and circRNA02370 were top up-regulated and circRNA05219 was top down-regulated DECs in SCI tissues. Furthermore, we predicted that mmu-miR-7056-5p, mmu-miR-5130, mmu-miR-2861, mmu-miR-6971-5p, mmu-miR-207, and mmu-miR-1956 were the downstream targets of circRNA02370. However, the study of circRNA02778, circRNA02370, and circRNA05219 in SCI remain a blank, whether they act as ceRNA to mediate the pathological process of SCI in a miRNA-dependent manner, which need further investigation.

Then we performed GO and KEGG function enrichment analysis on the host genes of DECs, and observed that Prkce (circRNA01429 host gene) and Nf1 (circRNA00537) were involved in the regulation of synaptic transmission, GABAergic and enzyme activator activity; Dock10(circRNA00105), Myo5a(circRNA03290), Ube3a(circRNA02860), and Epha3 (circRNA05219) were related to synapse organization; Bcl2l13(circRNA02778) linked to mitophagy; Myo5a associated with postsynaptic actin cytoskeleton and postsynaptic cytoskeleton; and Prkce linked to presynaptic cytosol and inflammatory mediator regulation of TRP channels. Consistent with our results, Shi et al (37). found that several DEGs participated in axon guidance, dopaminergic synapses, glutamatergic synapses, GABAergic synapses, and inflammatory signaling pathway in rats SCI model by RNA-sequencing. Furthermore, synaptic transmission, synapse remodeling, synapse reorganization and neuroinflammation have implicated in SCI (34, 38–40). Thus, we hypothesized that the DECs might be closely related to the pathological process of SCI.

Growing evidence have unveiled that neuronal inflammatory and immune responses are the pathomechanism of SCI (34, 39–43). Buzoianu-Anguiano and colleagues believed that the inflammatory and immune responses develop as follows: first is the immune cells such as neutrophils and macrophages secrete different cytokines and ROS, and followed by activating the chemokine CXCL10 and type II histocompatibility complex MCH-II, leading to a pro-inflammatory and immune microenvironment (40). Shi et al (37). identified that DEGs most enriched in immune response *via* Go analysis. Grassner et al (41). pointed to neutrophils and eosinophils were activated and infiltrated in SCI patients with infection, indicating that systemic immune imbalance after SCI can predict the risk of infection and the recovery of neurological function. Axon regeneration exerts a crucial role in promoting neurological recovery after SCI, and immune and inflammatory cells are involved in the regeneration of damaged neuronal axons (39, 42, 43). Horn et al (42). confirmed that the interactions between activated macrophages and injured neuronal axons play a central role in axonal retraction. Kurimoto et al (43). uncovered that neutrophils first respond in immune system after SCI, which can promote axonal regeneration induced by inflammation.

In this study, we identified top 9 IRGs (Ptprc, Fcgr2b, Ccl2, Tlr7, Cxcl10, Cd28, Il10ra, Cd68, and Tlr3) related to SCI and found they were significantly upregulated after SCI. Interestingly, Yan et al (44). also reported that the relative expression level of Ptprc and Fcgr2b were remarkably increased after SCI *via* PCR detection. Wang et al (45). demonstrated that CXCL10 was remarkably increased in SCI and had positive correlation with pro-inflammatory response. CXCL10 and CCL2 were reported to act as a predictors of increased infections after SCI (41). Toll-like receptors (TLRs) have the potential to modulate inflammatory and initiate immune responses (46, 47). Gucluler et al (47). reported that there was a toll like receptor-7 (TLR7) and TLR9-mediated innate immune dysfunction in SCI patients. TLR3 and TLR7 also promoted the secretion of the pro-inflammatory cytokines in the spinal cord, and contributing to inflammatory pain (46). Even though, the reason why the expression levels of the IRGs significantly increased and their functions in the subacute stage of SCI needs to be further identified. Furthermore, more experiments need to be performed to validate and understand how the IRGs mediated SCI *via* regulating neuronal inflammatory and immune responses.

Nevertheless, this study also has several limitations need to be taken seriously. First is the predictive results were mainly based on bioinformatics analysis, and the study samples were collected from rats rather than humans, so the relevant molecular biology and animal experiments were needed to further demonstrate the immunomodulatory effects and corresponding mechanisms of the key IRGs after SCI. Then there’s the relatively small size of the sample that might affect the conclusions. Finally, the correlation of circRNA, miRNA, and mRNA, and whether they constitute the circRNA-miRNA-mRNA ceRNA signaling axis that plays a role in SCI, is also a topic worthy of investigation.

## Conclusion

In summary, we screened 24 DECs and 372 DEGs by high-throughput RNA sequencing analysis, and found that circRNA02778, circRNA02370, and Ctsd were top up-regulated as well as circRNA05219 and Huwe1 were top down-regulated after SCI. Functional enrichment analysis shown that the functions of DECs are mainly focused on synaptic functions and inflammatory response. Meanwhile, we identified multiple IRGs (Ptprc, Fcgr2b, Ccl2, Tlr7, Cxcl10, Cd28, Il10ra, Cd68, and Tlr3) related to SCI and found they were significantly upregulated after SCI, but the specific mechanisms by which they regulate SCI by mediating neuronal inflammatory and immune responses need to be further explored. Findings of this study provide new direction and molecular basis for the diagnosis and treatment of SCI in the future.

## Data availability statement

The data presented in the study are deposited in the GEO repository (https://www.ncbi.nlm.nih.gov/gds/), accession number GSE217797. We declare that the data supporting the findings of this study are available with in the article and from the corresponding author upon reasonable request.

## Ethics statement

The animal study was reviewed and approved by Xuanwu Hospital ethics committee, Capital Medical University.

## Author contributions

YL designed and wrote the manuscript. XC and SL critically reviewed and revised the manuscript. BW and WS performed the bioinformatics analysis. GL and CK collected samples for RNA-sequencing. All authors contributed to the article and approved the submitted version.

## Funding

This research was funded by National key R&D plan(2020YFC2004905) R&D Program of Beijing Municipal Education Commission (KZ/KM/SZ/SM2022100250**), and Beijing Municipal Medical Science Institute-Public Welfare Development Reform Pilot Project (Capital Medical Research No. 2019-2).

## Conflict of interest

The authors declare that the research was conducted in the absence of any commercial or financial relationships that could be construed as a potential conflict of interest.

## Publisher’s note

All claims expressed in this article are solely those of the authors and do not necessarily represent those of their affiliated organizations, or those of the publisher, the editors and the reviewers. Any product that may be evaluated in this article, or claim that may be made by its manufacturer, is not guaranteed or endorsed by the publisher.
